# Deep Learning-Based Gender Recognition in Cherry Valley Ducks Through Sound Analysis

**DOI:** 10.3390/ani14203017

**Published:** 2024-10-18

**Authors:** Guofeng Han, Yujing Liu, Jiawen Cai, Enze Duan, Zefeng Shi, Shida Zhao, Lianfei Huo, Huixin Wang, Zongchun Bai

**Affiliations:** 1 Institute of Agricultural Facilities and Equipment, Jiangsu Academy of Agricultural Sciences, Nanjing 210014, China; hanguofeng@jaas.ac.cn (G.H.); liuyujing_work@163.com (Y.L.); renaissance6969@163.com (J.C.); 20220049@jaas.ac.cn (E.D.); 18115752826@163.com (Z.S.); 20220069@jaas.ac.cn (S.Z.); 20210044@jaas.ac.cn (L.H.); wanghuixin@jaas.ac.cn (H.W.); 2 Key Laboratory of Protected Agriculture Engineering in the Middle and Lower Reaches of Yangtze River, Ministry of Agriculture and Rural Affairs, Nanjing 210014, China

**Keywords:** gender identification, sound information, BP neural network, deep neural network, convolutional neural network

## Abstract

Gender recognition is an important part of the duck industry. Currently, the gender identification of ducks mainly relies on manual labor, which is highly labor-intensive. This study aims to propose a novel method for distinguishing between males and females based on the characteristic sound parameters for day-old ducks. The effective data from the sounds of day-old ducks were recorded and extracted using the endpoint detection method. The 12-dimensional Mel-frequency cepstral coefficients (MFCCs) with first-order and second-order difference coefficients in the effective sound signals of the ducks were calculated, and a total of 36-dimensional feature vectors were obtained. These data were used as input information to train three classification models, include a backpropagation neural network (BPNN), a deep neural network (DNN), and a convolutional neural network (CNN). The training results show that the accuracies of the BPNN, DNN, and CNN were 83.87%, 83.94%, and 84.15%, respectively, and that the three classification models could identify the sounds of male and female ducks. The prediction results showed that the prediction accuracies of the BPNN, DNN, and CNN were 93.33%, 91.67%, and 95.0%, respectively, which shows that the scheme for distinguishing between male and female ducks via sound had high accuracy. Moreover, the CNN demonstrated the best recognition effect. The method proposed in this study can provide some support for developing an efficient technique for gender identification in duck production.

## Simple Summary

In the duck breeding process, the rapid and accurate identification of gender is currently a key challenge. This study proposes a method based on sound signal discrimination to address this issue. The vocalizations of one-day-old male and female ducklings were extracted and the signal features were obtained through professional processing. Classification models were established using a backpropagation neural network (BPNN), a deep neural network (DNN), and a convolutional neural network (CNN). In total, 70% of the dataset was used for training, and 30% was used for testing. The test results showed that the BPNN model had the highest accuracy rate of 93.33% in identifying male ducks, while the CNN model had the highest accuracy rate of 95% in identifying female ducks. From the perspective of overall model performance, the CNN model performed the best, with the highest accuracy rate and F1-score of 84.15% and 84.32%, respectively. This study can provide a technical reference and a foundation for future voice recognition technologies for different poultry breeds and applications.

## 1. Introduction

Ducks, a kind of waterfowl, are an important commercial poultry species and an important source of meat worldwide. China is the global leader in both duck breeding and consumption. In recent years, the traditional small-scale free-range raising of livestock and poultry has begun to develop in the direction of large-scale, intelligent, and mechanized production [1]. Recently, the large-scale breeding model of the duck industry has progressed in China, and the requirement for efficient and intelligent techniques in duck production has become apparent. The growth rates and nutritional requirements of male and female ducks differ, resulting in inadequate utilization of feed nutrients, significant weight disparities, and a lack of standardization in the slaughter line. Thus, sex determination and subsequent separate breeding of meat ducks have become industry requirements for the future [2]. Here, the intelligent identification of male and female ducks is important. Currently, vent sexing, or venting, is often used to determine the gender of ducks. However, vent sexing needs to be completed within a certain period of time and causes stress due to the manual handling of the birds. This manual identification is labor-intensive, and the sexer only learns to identify the gender with regular practice [3,4].

Sound is an important parameter among the numerous phenotypic sources of information available for livestock and poultry, as it can reflect their health status, as well as other physiological and growth-related information [5]. With the rapid development of sound digital processing technology, many researchers have conducted work on the characteristic information of livestock and poultry vocalizations and have obtained numerous scientific research results [6,7,8,9,10,11]. Layer hen vocalization has been detected, and the identification and classification of various vocalizations of laying hens have also been completed under complex background sounds based on voice features [12]. The detection, identification, and classification of normal or abnormal vocalizations, coughing, and other kinds of sounds is also possible in pigs via sound analysis [13]. It is possible to carry out real-time monitoring of respiratory abnormalities after vocalization identification, classification, and feature recognition for cough sounds in sheep [14]. Moreover, the intelligent detection of the physiological and growth conditions of livestock and poultry is achieved through their vocal information, including disease identification, weight detection, pecking behavior detection in chickens, cough monitoring in pigs and cattle, various vocalization recognition classifications, etc. [15,16,17].

The sex of a duckling can be determined via its call, as recognized by an experienced breeder. One recent study also suggested that acoustic signal analysis could be a method for the automated sex identification of ducklings [18]. In recent years, artificial intelligence technology, including machine learning, deep learning, etc., has been applied to animal husbandry research and production. The results indicate that by utilizing the vocal information of livestock and poultry, supplemented with machine learning and deep learning algorithms, their vocal characteristics can be effectively identified. However, previous studies based on deep learning have mainly focused on the sound parameters of pigs, cattle, sheep, broilers, and laying hens. There are currently no relevant reports on research into deep learning-based sound analysis of waterfowl. The waterfowl breeding industry is important in China. Research on the sound information of waterfowl, including the meat-type duck, would be beneficial to its industrial development. Thus, this study aims to develop a method for gender identification in meat-type ducks based on sound analysis, which could achieve the accurate and intelligent identification of male and female ducks through sound information, providing theoretical and technical support for the automated and intelligent development of the breeding industry.

## 2. Materials and Methods

This study was performed according to the Research Committee of the Jiangsu Academy of Agricultural Sciences and was carried out in strict accordance with the Regulations for the Administration of Affairs Concerning Experimental Animals [Permit Number SYXK (Su) 2020-0024].

### 2.1. Experiment Design for Sound Collection

**Figure 1:**
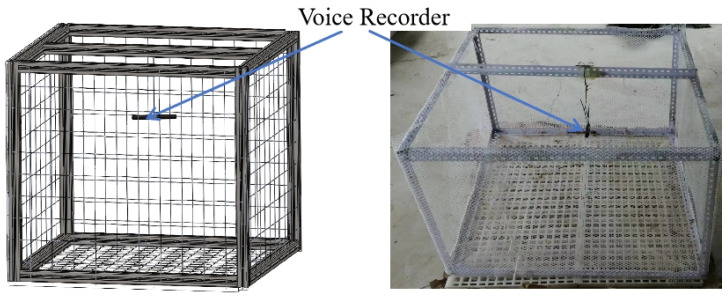
Layout for sound collection.

A total of 300 one-day-old Cherry Valley ducks, 150 males and 150 females, were purchased from a local commercial hatchery (Suqian Yike Breeding Poultry Co., Ltd., Jiangsu, China) for this experiment. The ducks were reared in an environmentally controlled poultry room in the animal center of the Jiangsu Academy of Agricultural Sciences. The experiment was conducted from May to June 2023. A breeding box (500 × 500 × 500 mm) was built and placed inside the poultry room. The sides and bottom of the box were covered with plastic mesh. In order to record the ducks’ voices clearly, the voice recorder (B618, Lenovo Corporation, Beijing, China) was set in the center of the rearing area, 400 mm from the ducks (Figure 1).

When the ducks were in a stable condition, the sounds of the ducks were recorded in turn for more than 2 min. During the recording process, 44,100 Hz 16-bit mono sampling parameters were set with the voice recorder, and the data of the ducks’ voices were saved in the WAV file format. 

When meat ducks are not in a life-threatening situation, their call frequency is concentrated around 2500 Hz. However, when the meat ducks detect a crisis situation, their call frequency is mainly concentrated between 2800 and 2900 Hz in order to convey the danger to their companions. At the same time, due to the sound resulting from the ducks stepping on the net while running and escaping, the recorded sounds also contained some signals below 1000 Hz. When ducks are in a dark environment, the frequency of the sounds they make to find their companions exceeds 3000 Hz, conveying a more urgent distress call.

The clear and non-overlapping sound clips of ducks were selected using audio processing software (Audacity, https://www.audacityteam.org/download/windows/, accessed on 13 October 2024). The sound dataset was obtained using audio processing, in which the sound data of 120 male ducks and 120 female ducks were randomly selected as the training set, while the sounds of the remaining 60 ducks were used as the testing set.

### 2.2. Prepossessing of Duck Sound Data

In order to obtain further information from the sound signals, it was necessary to preprocess them before research. The preprocessing of the sound signals mainly included three parts: namely, pre-emphasis, windowing and framing, and endpoint detection [19]. Next, we will introduce these three parts.

In the analysis of voice signals, the loss of sound signal energy caused by the propagation medium becomes more serious as the frequency of the sound signal increases. In order to compensate for the loss of the high-frequency part of the sound signal, the sound signal was optimized through a pre-emphasis digital filter. The formula is as follows:

(1) $$H(z)=1-\alpha z^{-1}$$

In this formula, *α* represents a pre-emphasis parameter, taken as 0.95 for this study.

Duck vocalization is a complex and unstable speech signal; however, within a relatively short period of time (10~30 ms), it can be considered to be in a stable or unchanged state. This study selected 25 ms as the length of a frame and then performed framing and windowing processing in order to reduce the impact of spectrum leakage and reduce the truncation effect of each frame of meat duck sound signals. In this study, the length of the Hamming window was selected for speech framing. The expression of the Hamming window function *w*(*n*) is shown in Formula (2) as follows:

(2) $$\omega (n)=\left\{\begin{array}{c} 0.54-0.46\cos(\frac{2\pi n}{N-1})\\ 0,\;others \end{array}\right.,\;0\leq n\leq N-1$$

In this formula, *N* represents the number of samples in the unit frame.

The starting and ending positions of the duck’s voice were marked through voice endpoint detection. The data marked as valuable were extracted, and the silent segments were discarded. The manually selected sound clips of the ducks’ voices were endpoint detected using a single-parameter dual-threshold endpoint detection algorithm. The calculation formulas [20] of the short-term energy *E*(*i*) and short-term zero-crossing rate *Z*(*i*) of the *i*-th frame speech signal are shown in Equations (3)–(5) as follows:

(3) $$E(i)=\sum_{n=0}^{L-1}x_{i}^{2}(n)$$

(4) $$Z(i)=\frac{1}{2}\sum_{n=0}^{L-1}\left|\mathrm{sgn}\left[x_{i}(n)\right]-\mathrm{sgn}\left[x_{i}(n-1)\right]\right|$$

(5) $$\mathrm{sgn}\left[x\right]=\left\{\begin{array}{c} 1,\;x\geq 0\\ -1,\;x<0 \end{array}\right.$$

In these formulas, *L* is the frame length, *x_i_*(*n*) is the value of the *n*-th sampling point of the *i*-th frame data, and sgn[] is the sign function.

The parameter *K_n_* was obtained through multiplying the short-term energy of the duck sounds by the value of the short-term zero-crossing rate. The parameter *K_n_* can be interpreted as the mixed characteristics of a frame of duck sounds, as shown in (6). *K_n_* was used as the input parameter of the endpoint detection algorithm, an appropriate threshold was selected, and the effective sound fragment information in the duck’s sounds was extracted. This method is called the zero-product endpoint detection method [21]. In the overall sound segment, there are both duck calls and environmental noise. By selecting the appropriate value of *K_n_* in this method, the noises with values below the *K_n_* parameter were identified as noise and removed, thereby achieving noise reduction in the sound.

(6) $$K_{n}=\frac{1}{2}\sum_{n=0}^{L-1}\left|\mathrm{sgn}\left[x_{i}(n)\right]-\mathrm{sgn}\left[x_{i}(n-1)\right]\right|\cdot \sum_{n=0}^{L-1}x_{i}^{2}(n)$$

**Figure 2:**
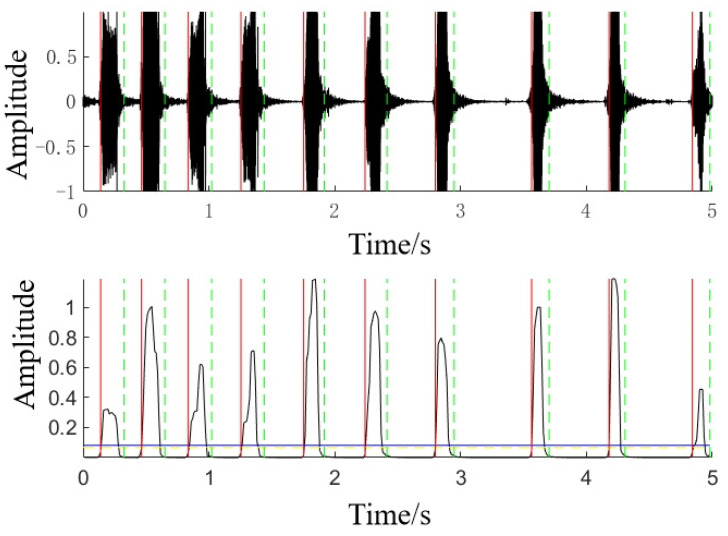
Endpoint detection of a duck’s vocalization. (The red solid line and green dashed line represent the starting and ending points of the signal, respectively).

The endpoint detection effect based on this method is shown in Figure 2, in which the solid red line represents the starting point of the duck’s vocalization and the green dotted line represents the end point of the duck’s vocalization.

### 2.3. Extraction of Characteristic Parameters of Duck Vocalizations

The meat duck sound signal obtained after preprocessing and speech enhancement, as shown above, is only a one-dimensional time series with no additional data features. It is necessary to extract features from the sound signals of meat ducks and normalize them before they can be used as input matrices for neural networks.

**Figure 3:**
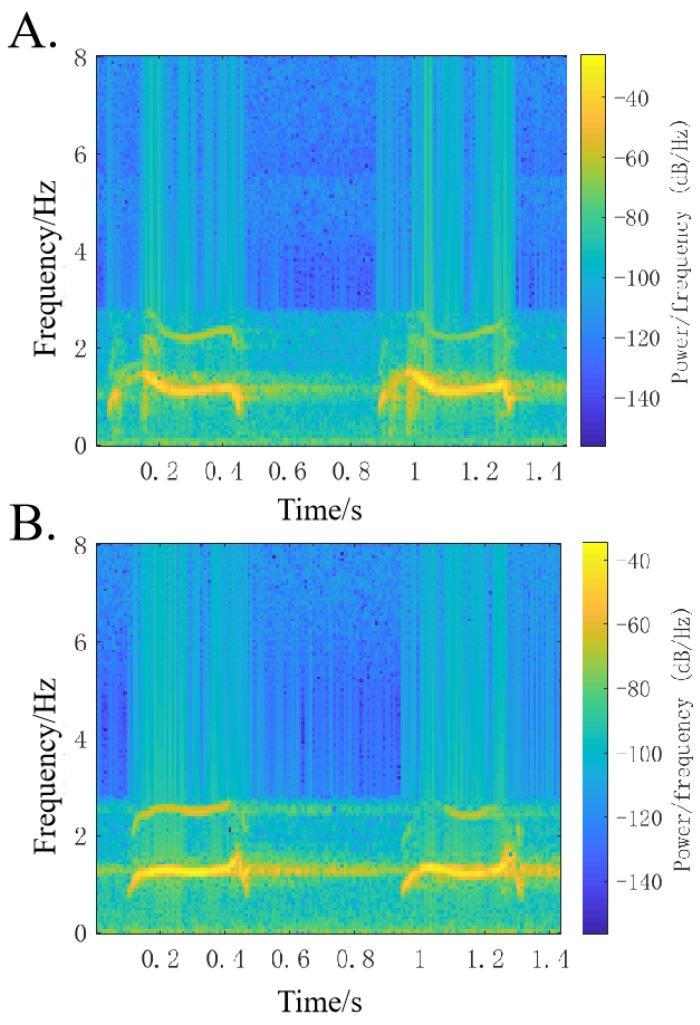
Spectrograms of vocalizations in male (**A**) and female (**B**) ducks.

In voice signal analysis, a spectrogram is an important part of the analysis process. It can convert the one-dimensional feature sequence of the sound into a visualized two-dimensional feature sequence and express the time domain and frequency domain information of the sound signal at the same time, which is highly intuitive [22]. Figure 3 shows a spectrogram of the vocalizations of male and female ducks. The harmonic curves of the male and female ducks have significant differences. The spectrogram of the female duck shows that during the vocalization process, the frequency of the entire sound band is relatively stable, only slightly increasing at the end. The spectrogram of the male duck, on the other hand, shows that its vocalization frequency is higher at the beginning, then gradually decreasing and stabilizing. These differences can be used to distinguish the call characteristics of male and female ducks. Based on this method, it is possible to distinguish male ducks from female ducks through sound. The information in ducks’ vocalizations contains a certain number of redundant signals, and it is necessary to extract the features from the sound information to reduce the random redundant information. Therefore, the extracted feature parameters are the key to later classification and recognition. 

**Figure 4:**
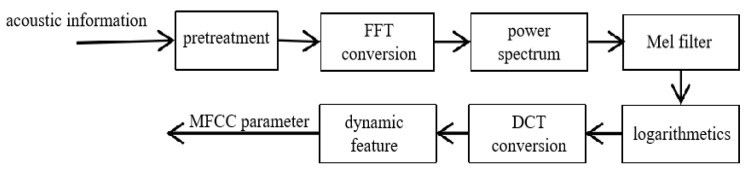
Flowchart of the Mel-frequency cepstral coefficient (MFCC) feature parameter extraction.

In sound processing, the Mel-frequency cepstrum (MFC) is a representation of the short-term power spectrum of a sound [23,24,25], based on a linear cosine transform of a log power spectrum on a nonlinear Mel scale of frequency. The Mel-frequency cepstral coefficients (MFCCs) are coefficients that collectively make up an MFC [26,27,28,29,30], which is established based on the human auditory mechanism. These MFCCs have good anti-noise and recognition performance and comprise an important feature vector in the process of voice signal analysis. The MFCC feature parameter extraction process is shown in Figure 4.

The preprocessing mainly included pre-emphasis and frame windowing, which have been introduced earlier and will not be further described.

After two small steps of processing, *x*(*n*) obtains the segmented sound signal *x_i_*(*m*), with subscript *i* representing the *i*-th frame. The second step is the Fast Fourier transform (FFT), which converts the time-domain sound signal *x_i_*(*m*) into a frequency-domain signal *X*(*i*,*k*) (subscript *i* represents the *i*-th frame, *k* represents the *k*-th spectral line), i.e., the frequency spectrum. The conversion formula is

(7) $$X(i,k)=FFT[x_{i}(m)]$$

The third step is to calculate the spectral line energy. For this, we take the modulus square of the spectral data obtained in the third step to obtain the spectral line energy of the sound signal. The calculation formula is shown in Equation (8):

(8) $$E(i,k)={\left|X(i,k)\right|}^{2}$$

The fourth step is to calculate the energy passing through the Mel filter. The spectral line energy obtained above is filtered through a set of Mel scale triangular filter banks, abbreviated as Mel filter banks. The frequency domain response *H_m_*(*k*) is shown in Equation (9), and the obtained energy *S*(*i*,*m*) is shown in Equation (10):

(9) $$H_{m}(k)=\left\{\begin{array}{l} 0\;\;\;\;\;\;\;\;\;\;\;\;\;\;\;\;\;\;\;\;\;\;\;\;\;\;\;\;\;\;\;\;k<f(m-1)\\ \frac{k-f(m-1)}{f(m)-f(m-1)}\;\;\;\;f(m-1)\leq k\leq f(m)\\ \frac{f(m+1)-k}{f(m+1)-f(m)}\;\;\;\;f(m)<k\leq f(m+1)\\ 0\;\;\;\;\;\;\;\;\;\;\;\;\;\;\;\;\;\;\;\;\;\;\;\;\;\;\;\;\;\;\;\;k>f(m+1) \end{array}\right.,\;0\leq m<M$$

(10) $$S(i,m)=\sum_{k=0}^{N-1}E(i,k)H_{m}(k),\;0\leq m<M$$

The fifth step is logarithmic operation and discrete cosine transform. After performing logarithmic operation on the Mel-filtered signal mentioned above and then performing discrete cosine transform, the cepstral signal of the original signal, i.e., the MFCC characteristic parameters, can be obtained via the following operation:

(11) $$mfcc(i,n)=\sqrt{\frac{2}{M}}\sum_{m=0}^{M-1}\log[S(i,m)]\cos[\frac{\pi n(2m-1)}{2M}]$$

**Figure 5:**
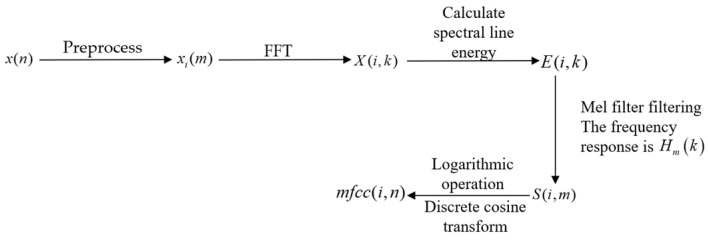
The calculation process of the Mel-frequency cepstral coefficient (MFCC) parameters.

After the above five steps, one can convert the time-domain signal into a frequency-domain signal. Cepstral signals can characterize the spectral characteristics of signals. A cepstral operation can weaken the correlation between features—that is, reduce the noise between dimensions. The overall calculation process is shown in Figure 5.

In practical applications of speech recognition, a large number of researchers often extract 12-dimensional MFCC feature parameters. However, the 12-dimensional MFCC feature parameters can only reflect the static spectral characteristics of speech. Therefore, researchers further extract the first-order and second-order differential coefficients of 12 dimensions through differential operations, while the 24-dimensional differential coefficients can capture the temporal changes and dynamic characteristics of speech signals.

### 2.4. Establishment of Classification Model for Male and Female Ducks

A BP neural network (BPNN) and a deep neural network (DNN) are both feed-forward neural networks [31]. Their main characteristics are signal forward propagation and error backpropagation. They are often used for regression fitting and recognition classification. There are usually a 3-layer network structure—including a signal input layer—a hidden layer, and a result output layer. The difference from the BP neural network topology lies in the hidden layer. A DNN contains more hidden layers. Therefore, a DNN can handle large-scale training samples.

In this study, there were three hidden layers in the BP neural network. The number of neurons in each layer was 18, 9, and 4, respectively, and each layer used the ReLU function as the activation function. The DNN had a total of 4 layers, and the number of neurons in each layer was 48, 32, 16, and 8, respectively. The activation functions of each layer were ReLU functions, and the dropout parameters were all 0.1. Then, the probability of each sample was calculated via the softmax function and used as the output result.

Additionally, the convolutional neural network (CNN) is a commonly used deep learning algorithm, widely used in image and speech recognition [32,33,34,35]. The CNN mainly consists of an input layer, a convolution layer, a pooling layer, and a fully connected layer. The CNN in this study had a total of 4 convolutional layers, each containing 128, 128, 64, and 32 convolution kernels, respectively. The operation of the convolution layer can extract more information. The convolution kernel of each layer used a 3*3 two-dimensional convolution kernel. The activation function of each convolutional layer was the ReLU function, and the loss rate was 0.3. Then, we used the fully connected layer to flatten it into one-dimensional data. Finally, the probability of each sample was calculated using the softmax function and was used as the output result.

### 2.5. Model Evaluation Method

This study aimed to determine the gender of Cherry Valley ducks via sound analysis. The results of the classification model were scored based on the actual sex and the model-predicted sex of each duck. The confusion matrix is the most commonly used model evaluation index in deep learning, and its composition is shown in Table 1. The confusion matrix can be used to extract secondary evaluation indicators. The accuracy, recall, specificity, and F1-score were used as model evaluation indicators in this study, and their calculation expressions are shown below (Equations (12)–(16)). Moreover, the recall represents the recognition accuracy for male ducks, and the specificity represents the recognition accuracy for female ducks.

To achieve more representative results, we implemented 5-fold cross-validation to evaluate the method’s performance. The chicks were divided into five groups, allowing the model to undergo training and testing five times. Four groups were designated for training the model, while the fifth group served as the testing set. The final result was determined by calculating the average outcome from the five experiments [36].

(12) $$\mathrm{Accuracy}(A)=\frac{\left(TP+TN\right)}{\left(TP+TN+FP+FN\right)}$$

(13) $$Recall(R)=\frac{TP}{\left(TP+FN\right)}$$

(14) $$Specificity(S)=\frac{TN}{\left(FP+TN\right)}$$

(15) $$Precision(P)=\frac{TP}{\left(TP+FP\right)}$$

(16) $$F1\text{-}score=2*\frac{P*R}{P+R}$$

## 3. Results and Discussion

**Figure 6:**
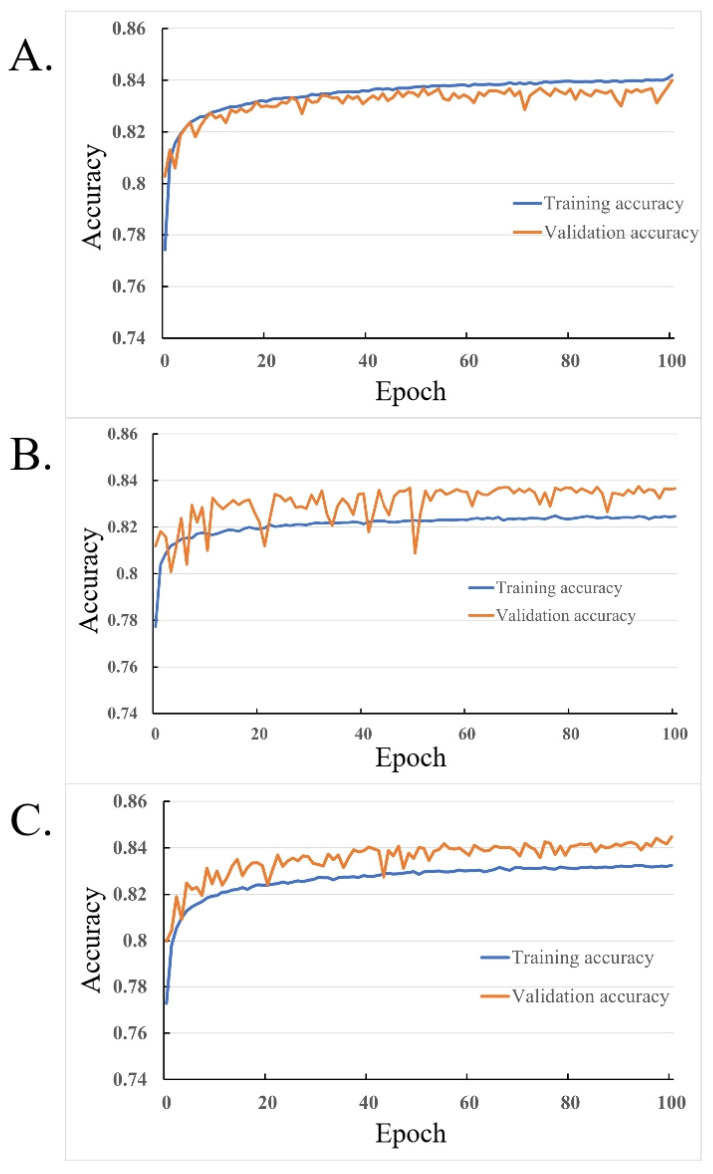
Training accuracy and validation accuracy of the three methods: (**A**) BPNN, (**B**) DNN, and (**C**) CNN. BPNN, backpropagation neural network; DNN, deep neural network; CNN, convolutional neural network.

**Figure 7:**
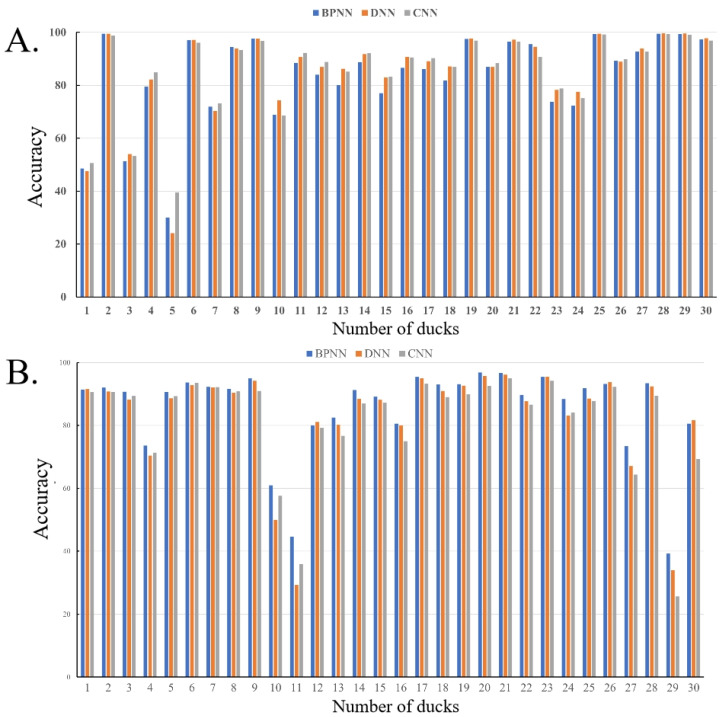
Gender prediction results for 60 meat ducks. (**A**) represents 30 male ducks, and (**B**) represents 30 female ducks. BPNN, backpropagation neural network; DNN, deep neural network; CNN, convolutional neural network.

The sounds of 300 Cherry Valley ducks, including 150 male and 150 female ducks, were recorded and analyzed in this study. The sounds of 240 ducks were used to train the classification model, and the sounds of 60 ducks were used to detect the classification model. The endpoints of the ducks’ sounds were determined through the endpoint detection method. In order to balance the dataset, 3000 sound frames were extracted from each meat duck, for a total of 720,000 sound frames to train the classification model and 180,000 sound frames to detect the classification model. Among the sound frames of the training dataset, 70% were used as the training set, 15% were used as the verification set, and the other 15% were used as the testing set. Each sample sound frame was composed of a 36-dimensional feature vector, including a 12-dimensional MFCC and its first-order and second-order difference coefficients. A BP neural network, a DNN, and a CNN were constructed to identify and classify male and female duck vocalizations, and each classification model was iterated 100 times. After model training and testing, the test results of the classification model were obtained, as shown in Table 2. The training process is shown in Figure 6. The accuracy of the classification and recognition of the calls of each male and female duck is shown in Figure 7. The trained classification model was applied to the remaining 60 broiler ducks, and the three types of predicted results are shown in Table 3.

As shown in Figure 6, the training process of the three classification models was as follows: with the increase in the training iterations, the training accuracy of the three models all exceeded 82%, and the validation accuracy all exceeded 83%. Since there was no testing set in this experiment, with only training and validation sets, the fact that the validation accuracy was higher than the training accuracy indicates that the models were correct.

However, the BPNN model had a lower validation accuracy than the training accuracy. This is likely because the BPNN structure was too simple, and it lacked the robustness of the CNN and DNN models when the dataset was small.

By observing the BPNN graph, we can see that its structure was much simpler compared to the DNN and CNN. As the most basic neural network model, the BPNN’s loss function may have multiple local minima, leading to oscillations or becoming stuck in local optima during the training process. Therefore, the convergence and stability of the BPNN were worse than those of the DNN and CNN, which also caused the final validation accuracy, shown in Figure 6A, for the BPNN to have relatively large fluctuations.

The test results for the three trained classifications of the dataset are shown in Table 2. From the perspective of the ducks’ gender determination, the BPNN accurately predicted male ducks, with a recognition rate of 93.33%, while for the identification and classification of female ducks, the CNN had better accuracy, with a recognition rate of 95%. From the perspective of the entire model, the CNN had the highest accuracy and F1-score, reaching 84.15% and 84.32%, respectively. Therefore, it could be initially judged that the CNN classification model was better than the other two models.

After the model training was completed, the optimal types of the duck gender identification models were obtained, and the gender of the remaining 60 meat ducks was predicted, including 30 male ducks and 30 female ducks. According to the real gender, the prediction results of the three classification models are shown in Table 3. The prediction results of the three classification models for 30 male ducks were 28, 28, and 29, respectively. The prediction results for 30 female ducks were 28, 27, and 28, respectively. Overall, the recognition rates of the three classification models for male ducks were higher than those for female ducks. Among them, the BPNN accurately recognized 56 out of the 60 broiler ducks, with an accuracy rate of 93.33%. The DNN recognized 55 ducks, with an accuracy rate of 91.67%. The CNN recognized 57 ducks, with an accuracy rate of 95%. For male ducks, the recognition accuracy of the three classification models for 27 male ducks reached 60%, and the recognition accuracy for 23 male ducks was more than 80%. However, the recognition accuracy of the three male ducks with serial numbers 1, 3, and 5 was low. For the 30 female ducks in the test set, the predicted recognition accuracy of 27 female ducks was over 60%, and the recognition accuracy of 25 ducks reached 80%, while the CNN recognition accuracy was relatively lower. Moreover, for the three female ducks numbered 10, 11, and 29, the recognition accuracy was lower than 60%, which might be due to errors in the manual judgment process; that is, there were mistakes in identifying the gender of ducks during the manual verification process after the algorithm detection, which led to a lower recognition accuracy rate of the reference samples, resulting in the low identification accuracy of the reference sample.

## 4. Conclusions

This study used sound recognition technology and deep learning methods to extract the voice information of 300 one-day-old ducks, of which 240 were used as the training set and 60 were used as the testing set. For each duck, we extracted 36-dimensional characteristic parameters and three different models to identify the gender of ducks based on sound signals. The BPNN, DNN, and CNN classification models were able to identify the gender of the ducks via sound signals, and the recognition accuracy of the three models was over 83%. Among them, the BPNN’s recognition accuracy for male ducks was higher than the other two classification models, while the CNN’s recognition accuracy for female ducks was higher than the other two classification models. Overall, the CNN recognition classification model F1-score reached 84.32%, with the best recognition result.

The three models all made incorrect predictions on the same duckling individuals; however, more ducklings had their gender correctly identified. After comparing the various results, the CNN model achieved the best performance in using duckling vocalizations to determine gender, with the smallest prediction error for the ducklings. The three trend graphs in Figure 6 show that compared to the BPNN, the CNN had stronger robustness in low-sample and simple structures and did not exhibit overfitting like the BPNN. Compared to the images of the DNN, the CNN also had higher-quality curves with smaller fluctuations and was more stable. Therefore, compared to the BPNN and DNN, the CNN had greater advantages in classification.

The method proposed in this paper can predict the gender of chicks based on their vocalizations, and the accuracy and efficiency of the model can be further improved in the future. A single model using CNN can be adopted, but to combine the advantages of different algorithms, a hybrid approach combining DNN and CNN needs to be examined. The CNN can be used for feature extraction, and its output can be used as the input to the DNN, which can then perform the classification and regression tasks. Alternatively, convolutional and pooling layers can be inserted into the hidden layers of the DNN, forming a mixed topological structure. By combining the advantages of different algorithms, accuracy and efficiency can be improved. However, each algorithm needs to consider the practical application, and a universally applicable algorithm design is not yet achievable. Based on different breeds and recognition requirements, different algorithm model systems should be designed and improved in real-time.

## Figures and Tables

**Table 1:** Evaluation indices of the model.

	Positive Condition	Negative Condition
Positive Prediction	True Positive (*TP*)	False Positive (*FP*)
Negative Prediction	False Negative (*FN*)	True Negative (*TN*)

**Table 2:** Three kinds of classification model test results.

	BPNN	DNN	CNN
Accuracy (%)	83.87	83.94	84.15
Recall (%)	84.60	83.20	83.52
Specificity (%)	83.17	84.71	84.80
F1-score (%)	83.72	84.07	84.32

**Table 3:** Prediction results of the three classification models.

	BPNN	DNN	CNN
Male (n = 30)	28	28	29
Female (n = 30)	28	27	28
Recognition accuracy (%)	93.33	91.67	95.00

## Data Availability

Data are contained within the article.
